# Clinical findings of patients with coronavirus disease 2019 in Jiangsu province, China: A retrospective, multi-center study

**DOI:** 10.1371/journal.pntd.0008280

**Published:** 2020-05-08

**Authors:** Rui Huang, Li Zhu, Leyang Xue, Longgen Liu, Xuebing Yan, Jian Wang, Biao Zhang, Tianmin Xu, Fang Ji, Yun Zhao, Juan Cheng, Yinling Wang, Huaping Shao, Shuqin Hong, Qi Cao, Chunyang Li, Xiang-an Zhao, Lei Zou, Dawen Sang, Haiyan Zhao, Xinying Guan, Xiaobing Chen, Chun Shan, Juan Xia, Yuxin Chen, Xiaomin Yan, Jie Wei, Chuanwu Zhu, Chao Wu

**Affiliations:** 1 Department of Infectious Diseases, Nanjing Drum Tower Hospital, The Affiliated Hospital of Nanjing University Medical School, Nanjing, China; 2 Department of Infectious Diseases, The Affiliated Infectious Diseases Hospital of Soochow University, Suzhou, China; 3 Department of Critical Medicine, Huai'an No. 4 People's Hospital, Huai’an, China; 4 Department of Infectious Diseases, The Third People's Hospital of Changzhou, Changzhou, China; 5 Department of Infectious Diseases, Affiliated Hospital of Xuzhou Medical University, Xuzhou, China; 6 Department of Quality Control Office, Huai'an No. 4 People's Hospital, Huai’an, China; 7 Department of Infectious Diseases, The Third People’s Hospital of Yangzhou, Yangzhou, China; 8 Department of Infectious Diseases, Yancheng Second People’s Hospital, Yancheng, China; 9 Department of Infectious Diseases, The People’s Hospital of Suqian, Suqian, China; 10 Nursing Department, Huai'an No. 4 People's Hospital, Huai’an, China; 11 Department of Gastroenterology, Northern Jiangsu People’s Hospital, Clinical Medical College of Yangzhou University, Yangzhou, China; 12 Department of Neurology, The Affiliated Hospital of Kangda College of Nanjing Medical University, The First People's Hospital of Lianyungang, Lianyungang, China; 13 Department of Emergency, The Affiliated Hospital of Kangda College of Nanjing Medical University, The First People's Hospital of Lianyungang, Lianyungang, China; 14 Department of Infectious Diseases, Zhongda Hospital Southeast University, Nanjing, China; 15 Department of Laboratory Medicine, Nanjing Drum Tower Hospital, The Affiliated Hospital of Nanjing University Medical School, Nanjing, China; 16 Department of Infectious Diseases, Nanjing Drum Tower Hospital Clinical College of Nanjing Medical University, Nanjing, China; Universidade Federal de Minas Gerais, BRAZIL

## Abstract

Limited data are available for clinical characteristics of patients with coronavirus disease 2019 (COVID-19) outside Wuhan. This study aimed to describe the clinical characteristics of COVID-19 and identify the risk factors for severe illness of COVID-19 in Jiangsu province, China. Clinical data of hospitalized COVID-19 patients were retrospectively collected in 8 hospitals from 8 cities of Jiangsu province, China. Clinical findings of COVID-19 patients were described and risk factors for severe illness of COVID-19 were analyzed. By Feb 10, 2020, 202 hospitalized patients with COVID-19 were enrolled. The median age of patients was 44.0 years (interquartile range, 33.0–54.0). 55 (27.2%) patients had comorbidities. At the onset of illness, the common symptoms were fever (156 [77.2%]) and cough (120 [59.4%]). 66 (32.7%) patients had lymphopenia. 193 (95.5%) patients had abnormal radiological findings. 11 (5.4%) patients were admitted to the intensive care unit and none of the patients died. 23 (11.4%) patients had severe illness. Severe illness of COVID-19 was independently associated with body mass index (BMI) ≥ 28 kg/m^2^ (odds ratio [OR], 9.219; 95% confidence interval [CI], 2.731 to 31.126; P<0.001) and a known history of type 2 diabetes (OR, 4.326; 95% CI, 1.059 to 17.668; P = 0.041). In this case series in Jiangsu Province, COVID-19 patients had less severe symptoms and had better outcomes than the initial COVID-19 patients in Wuhan. The BMI ≥ 28 kg/m^2^ and a known history of type 2 diabetes were independent risk factors of severe illness in patients with COVID-19.

## Author summary

We reported the epidemiology, clinical laboratory, radiological characteristics, treatment, and clinical outcomes of 202 confirmed cases of COVID-19 in 8 hospitals from 8 cities of Jiangsu province, China. In this case series in Jiangsu Province, COVID-19 patients had less severe symptoms and had better outcomes than the initial COVID-19 patients in Wuhan. The BMI ≥ 28 kg/m^2^ and a known history of type 2 diabetes were independent risk factors of severe illness in patients with COVID-19. Sustained intensive control efforts remain urgently needed to minimize the risk of COVID-19.

## Introduction

During December 2019, severe acute respiratory syndrome coronavirus 2 (SARS-CoV-2) emerged in Wuhan, China and spread among humans in China and other countries [1,2]. Global attention was soon focused on this virus due to the rapidly increasing number of confirmed cases [3]. SARS-CoV-2 infection may result in severe and even fatal respiratory diseases [4]. As of March 13, 2020, 132,536 confirmed cases have been reported in 123 countries with 4,947 deaths [5]. Despite the rapid spread of the disease worldwide, the clinical characteristics of coronavirus disease 2019 (COVID-19) remain largely unclear. Furthermore, there are no directly antiviral drugs for COVID-19.

Several studies have reported the clinical characteristics of COVID-19 patients who were hospitalized in Wuhan (the outbreak center of the infection) [4,6,7]. Huang et al. first reported 41 cases of COVID-19 and most patients had a history of exposure to Huanan Seafood Market in Wuhan [6]. Fever and cough were the most common symptoms [6]. 13 (32%) patients were admitted to an intensive care unit (ICU) due to the severity of disease and six (15%) patients died [6]. Chen et al. conducted a retrospective, single-center study which included 99 confirmed cases of COVID-19 in Wuhan and found that the virus was more likely to infect older men with comorbidities, and the mortality rate was as high as 11% [4]. Another single-center study which analyzed 138 hospitalized patients with confirmed COVID-19 in Wuhan, found that 26% of patients received ICU care and the mortality rate was only 4.3% [7].

Although several studies have reported the clinical manifestations and short-term prognosis of COVID-19, the source of cases in majority of these studies was from a single center in Wuhan. The characteristics of the disease and risk factors of severe illness among inpatients in other parts of China outside Wuhan were still lacking. Chang et al. reported early clinical features of 13 patients with confirmed COVID-19 who were admitted to hospitals in Beijing, China [8]. In their study, all the patients recovered indicating milder clinical presentation caused by infections [8]. However, the sample size was very small with only inclusion of 13 patients and the study was limited by the lack of detailed data since patients were transferred to the designated hospitals after diagnosis [8]. Xu et al. summarized the clinical characteristics of 62 patients in Zhejiang province, which revealed that the symptoms of patients were relatively mild [9]. However, only 62 patients were included in the study and risk factors for severe illness could not be analyzed due to the limitation of sample size [9]. Nevertheless, these studies provide important evidence that the clinical characteristics of patients outside Wuhan may differ from those initially reported in Wuhan.

In this multi-center study, we aimed to describe the clinical characteristics of COVID-19 and to identify the risk factors of severe illness among inpatients with confirmed COVID-19 in Jiangsu province, which is located in the east of China.

## Methods

### Population

**Fig 1:**
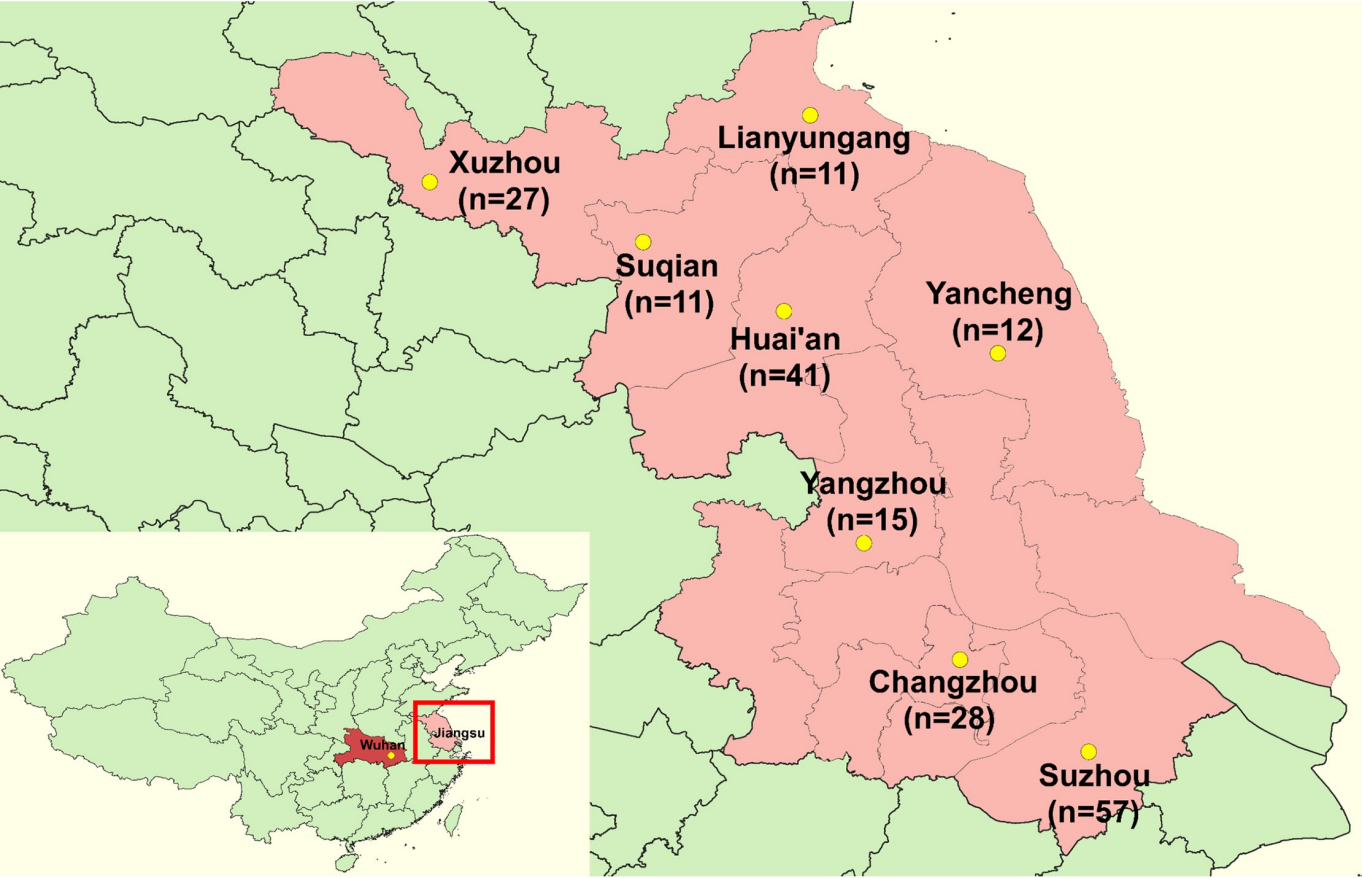
Geographical distribution of enrolled cases of coronavirus disease 2019 in Jiangsu, China.

This multi-center study retrospectively recruited 202 confirmed COVID-19 patients from 8 designated hospitals in 8 cities of Jiangsu province, China (Affiliated Hospital of Xuzhou Medical University [27 cases]; The Affiliated Hospital of Kangda College of Nanjing Medical University, The First People's Hospital of Lianyungang [11 cases]; Infectious Disease Hospital of Suqian [11 cases]; Huai'an No. 4 People's Hospital [41 cases]; Yancheng Second People’s Hospital [12 cases]; The Third People’s Hospital of Yangzhou [15 cases]; The Third People's Hospital of Changzhou [28 cases] and The Affiliated Infectious Diseases Hospital of Soochow University [57 cases]) (Fig 1).

Data of the confirmed COVID-19 patients were collected from January 22, 2020 to February 10, 2020. The 8 designated hospitals are responsible for the treatment of COVID-19 patients in Jiangsu province, China, assigned by the Chinese government. All confirmed patients were diagnosed based on the criterion of World Health Organization (WHO) interim guidance [10]. The study was approved by the institutional ethics board of these hospitals, with a waiver of informed consent.

### Procedures

All the medical records of confirmed cases were reviewed by more than two health care workers in each medical center. The data of epidemiology, clinical laboratory, radiology, treatment, and prognosis were collected from medical records. The demographic characteristics, comorbidities, exposure history, symptoms, signs, laboratory data, radiological data, treatment data, and outcomes were collected. All data were entered into a computerized database for further analysis. Different researchers preformed the cross check of the data to avoid errors. Unclear information was further clarified by directly contacting the specific clinicians who were responsible for the patients. The diagnostic criteria of acute respiratory distress syndrome (ARDS), acute cardiac injury, acute renal injury and acute liver injury were based on the corresponding guidelines [11–14]. Additionally, treatment medication and outcomes of the enrolled cases were also collected based on the medical records. The criteria for discharge was based on the guidelines for the diagnosis and treatment of novel coronavirus infection by the Chinese National Health Commission (Trial Version 5) [15]. Patients were grouped into severe and non-severe COVID-19 according to the same guidelines [15].

### Laboratory and radiological information

All patients were confirmed by throat swab sample obtained from the upper respiratory tract and detected by a real-time reverse transcriptase polymerase chain reaction (RT-PCR) in accordance with the protocol by the World Health Organization [16].The positive throat swab samples were confirmed by both hospitals and local centers for disease control and prevention. These routine laboratory tests were detected according to the state of disease, including blood routine tests, liver function, renal function, coagulation function, inflammatory biomarkers and myocardial enzymes. Radiological assessments including chest X-ray or chest computed tomography (CT) were performed for each patient.

### Statistical analysis

Continuous variables were described as medians (interquartile range (IQR)), and categorical variables were presented as the counts and percentages. Continuous variables were compared by the independent group t tests (normal distribution) and Mann-Whitney U (non-normal distribution). Categorical variables were compared by Chi-square or Fisher exact test. The risk factors for severe illness were analyzed by binary logistic regression. Variables having P values <0.05 in the univariate analysis were further used for a multivariate logistic regression analysis. P<0.05 was regarded to be statistically significant. The data analysis was performed by SPSS version 22.0 software (SPSS Inc., Chicago, IL, United States). Distribution map was plotted by QGIS version 3.10.2 (QGIS Development Team, Open Source Geospatial Foundation Project).

## Results

### Demographic and epidemiologic characteristics

A total of 202 admitted patients who were identified as COVID-19 were included in the study. The median age of the patients was 44.0 (IQR 33.0–54.0) years (Table 1). Six (3.0%) patients were children or adolescents. About half (57.4%) of patients were men. The median body mass index (BMI) was 24.4 kg/m^2^. Fifty-five (27.2%) patients had at least one underlying disease including hypertension (29 [14.4%]), type 2 diabetes (19 [9.4%]), chronic lung diseases (7 [3.5%]), chronic liver diseases (4 [2.0%]), cardiovascular diseases (5 [2.5%]), cerebrovascular diseases (3 [1.5%]), and malignant tumors (2 [1.0%]).

**Table 1:** Demographic and epidemiologic characteristics of patients with coronavirus disease 2019.

Variables (n [%] or median [IQR])	All patients (n = 202)	Non-severe patients (n = 179)	Severe patients (n = 23)	P value
Age (yr)	44.0 (33.0, 54.0)	44.0 (33.0, 53.0)	49.0 (35.0, 59.0)	0.066
Age range				0.185
≤30	36 (17.8)	35 (19.6)	1 (4.3)	
31–40	50 (24.8)	43 (24.0)	7 (30.4)	
41–50	53 (26.2)	49 (27.4)	4 (17.4)	
51–60	37 (18.3)	31 (17.3)	6 (26.1)	
≥61	26 (12.9)	21 (11.7)	5 (21.7)	
Gender				0.089
Male	116 (57.4)	99 (55.3)	17 (73.9)	
Female	86 (42.6)	80 (44.7)	6 (26.1)	
BMI (kg/m^2^) ^a^	24.4 (22.3, 26.4)	24.2 (22.1, 26.1)	26.4 (23.7, 29.5)	0.004
BMI range ^a^				<0.001
<28	148 (86.0)	138 (89.6)	10 (55.6)	
≥28	24 (14.0)	16 (10.4)	8 (44.4)	
**Comorbidities**				
Any comorbidity	55 (27.2)	46 (25.7)	9 (39.1)	0.173
Hypertension	29 (14.4)	27 (15.1)	2 (8.7)	0.411
Type 2 diabetes	19 (9.4)	11 (6.1)	8 (34.8)	<0.001
Chronic lung diseases	7 (3.5)	6 (3.3)	1 (4.3)	0.806
Chronic liver diseases	4 (2.0)	4 (2.2)	0 (0)	0.469
Cardiovascular diseases	5 (2.5)	4 (2.2)	1 (4.3)	0.539
Cerebrovascular diseases	3 (1.5)	2 (1.1)	1 (4.3)	0.228
Malignant tumors	2 (1.0)	2 (1.1)	0 (0)	0.610
Smoking history	16 (7.9)	14 (7.8)	2 (8.7)	0.884
**Exposure history** ^**b**^				
Contact with suspected or confirmed patient	98 (48.5)	92 (51.4)	6 (26.1)	0.022
Contacted with people from Wuhan or non-Wuhan areas of Hubei province	92 (45.5)	79 (44.1)	13 (56.5)	0.261
Visited Wuhan or non-Wuhan areas of Hubei province	84 (41.6)	73 (40.8)	11 (47.8)	0.519
Direct exposure to Huanan Seafood Market	3 (1.5)	3 (1.7)	0 (0)	0.532
No contact with Wuhan or non-Wuhan areas of Hubei Province	32 (15.8)	29 (16.2)	3 (13.0)	0.696
Time from symptom onset to admission (days)	5.0 (2.0, 7.0)	5.0 (2.0, 7.0)	5.0 (3.0, 9.0)	0.394

^a^ Available for 172 patients: 154 non-severe patients and 18 severe patients.

^b^ Within two weeks of symptom onset.

IQR, interquartile range; BMI, body mass index.

Although only 3 (1.5%) patients had direct exposure to Huanan Seafood Market, 84 (41.6%) patients visited Hubei province and 92 (45.5%) patients had contact with people who visited Hubei province after the onset of the COVID-19 epidemic. 98 (48.5%) patients had known contact with suspected or confirmed cases. However, 32 (15.8%) patients did not report any known contact with Hubei. The median time from symptom onset to admission was 5.0 days (IQR 2.0–7.0 days).

23 (11.4%) of the patients developed severe illness in our study. The age of severe patients was comparable with non-severe patients (median age 49.0 yr vs. 44.0 yr, P = 0.066) and the BMI index of severe patients was higher than non-severe patients (median BMI, 26.4 kg/m^2^ vs. 24.2 kg/m^2^, P = 0.004). Nearly half (44.4%) of the severe patients were obese (BMI ≥ 28 kg/m^2^) compared to only 10.4% of the non-severe patients. Furthermore, more severe patients had history of type 2 diabetes (34.8% vs. 6.1%, P<0.001). More non-severe patients contacted with suspected or confirmed patients than severe patients (51.4% vs. 26.1%, P = 0.022).

### Clinical features and laboratory abnormalities

The most common symptoms at onset of illness were fever (156 [77.2%]), cough (120 [59.4%]), followed by fatigue (44 [21.8%]), sore throat (24 [11.9%]), muscle ache (21 [10.4%]), shortness of breath (19 [9.4%]), diarrhea (13 [6.4%]), and headache (12 [5.9%]) (Table 2).

**Table 2:** Clinical characteristics and laboratory findings of patients with coronavirus disease 2019.

Variables (n [%] or median [IQR])	All patients (n = 202)	Non-severe patients (n = 179)	Severe patients (n = 23)	P value
**Onset symptoms**				
Fever	156 (77.2)	136 (76.0)	20 (87.0)	0.237
Cough	120 (59.4)	103 (57.5)	17 (73.9)	0.132
Fatigue	44 (21.8)	37 (20.7)	7 (30.4)	0.286
Sore throat	24 (11.9)	20 (11.1)	4 (17.4)	0.386
Muscle ache	21 (10.4)	18 (10.1)	3 (13.0)	0.659
Shortness of breath	19 (9.4)	13 (7.3)	6 (26.1)	0.004
Diarrhea	13 (6.4)	13 (7.3)	0 (0)	0.182
Headache	12 (5.9)	12 (6.7)	0 (0)	0.200
Rhinorrhoea	6 (3.0)	6 (3.4)	0 (0)	0.373
Vomiting	4 (2.0)	3 (1.7)	1 (4.3)	0.387
**Laboratory parameters** WBC (×10^9^/L)	4.5 (3.8, 5.7)	4.5 (3.7, 5.4)	5.6 (4.0, 6.9)	0.013
WBC range				0.809
No decreased	145 (71.8)	128 (71.5)	17 (73.9)	
Decreased	57 (28.2)	51 (28.5)	6 (26.1)	
Neutrophils (×10^9^/L)	2.8 (2.1, 3.8)	2.8 (2.1, 3.6)	4.5 (2.8, 5.9)	<0.001
Neutrophil range				0.388
No decreased	162 (80.2)	142 (79.3)	20 (87.0)	
Decreased	40 (19.8)	37 (20.7)	3 (13.0)	
Lymphocyte (×10^9^/L)	1.1 (0.8, 1.6)	1.2 (0.9, 1.6)	0.8 (0.5, 1.0)	<0.001
Lymphocyte range				0.100
No decreased	136 (67.4)	124 (69.3)	12 (52.2)	
Decreased	66 (32.7)	55 (30.7)	11 (47.8)	
Hb (g/L)	140.0 (128.0, 152.0)	140.0 (129.0, 153.0)	140.0 (127.0, 147.0)	0.628
PLT (×10^9^/L)	172.0 (133.5, 226.3)	172.0 (134.0, 228.0)	174.0 (132.0, 196.0)	0.562
Tbil (μmol/L)	9.9 (7.0, 14.0)	9.9 (6.8, 14.0)	9.8 (7.1, 15.4)	0.768
ALT (U/L)	25.0 (19.0, 35.0)	25.0 (18.0, 35.0)	31.0 (24.5, 51.0)	0.009
LDH (U/L)	236.5 (175.8, 370.3)	226.0 (173.6, 357.0)	368.5 (247.5, 638.0)	<0.001
ALB (g/L)	40.9 (38.0, 44.3)	41.1 (38.0, 44.5)	38.1 (33.7, 41.4)	0.002
GLB (g/L)	28.4 (25.0, 31.2)	28.5 (24.9, 31.2)	28.0 (26.3, 33.1)	0.492
Cr (μmol/L)	66.1 (54.9, 78.0)	64.0 (54.2, 76.4)	79.1 (69.0, 86.4)	0.005
Glu (mmol/L)	5.7 (5.1, 6.7)	5.7 (5.1, 6.6)	6.5 (5.5, 8.0)	0.009
Na (mmol/L)	139.0 (136.0, 141.0)	139.0 (136.1, 141.2)	137.7 (134.8, 141.0)	0.447
K (mmol/L)	4.0 (3.7, 4.4)	4.0 (3.7, 4.4)	3.9 (3.7, 4.4)	0.512
PT (s)	12.8 (12.0, 13.4)	12.8 (12.1, 13.4)	12.3 (11.7, 13.0)	0.089
PCT (ng/ml) ^a^				0.059
<0.05	87 (65.9)	78 (69.0)	9 (47.4)	
0.05–0.1	30 (22.7)	25 (22.1)	5 (26.3)	
≥0.1	15 (11.4)	10 (8.8)	5 (26.3)	
CRP (mg/L) ^b^				<0.001
<10	89 (61.8)	81 (65.3)	8 (40.0)	
10–50	44 (30.6)	38 (30.6)	6 (30.0)	
≥50	11 (7.6)	5 (4.0)	6 (30.0)	
cTnI ^c^				
No increased	101 (98.1)	87 (97.8)	14 (100.0)	0.571
Increased	2 (1.9)	2 (2.2)	0 (0)	
**Radiological findings**				
Chest CT				
No pneumonia	9 (4.5)	9 (5.0)	0 (0)	0.271
Unilateral pneumonia	35 (17.3)	34 (19.0)	1 (4.3)	0.081
Bilateral pneumonia	158 (78.2)	136 (76.0)	22 (95.7)	0.031
Ground glass opacity	149 (73.8)	129 (72.1)	20 (87.0)	0.127

^a^ Available for 132 patients: 113 non-severe patients and 19 severe patients

^b^ Available for 144 patients: 124 non-severe patients and 20 severe patients

^c^ Available for 103 patients: 99 non-severe patients and 14 severe patients

IQR, interquartile range; WBC, white blood cells; Hb, hemoglobin; PLT, platelet; Tbil, total bilirubin; ALT, alanine transaminase; LDH, lactate dehydrogenase; ALB, albumin; GLB, globulin; CR, creatinine; Glu, glucose; PT, prothrombin time; PCT, procalcitonin; CRP, C-reactive protein; cTnI, cardiac troponin I.

The blood counts of patients on admission showed leukopenia (57 [28.2%]) and lymphopenia (66 [32.7%]). 87 (65.9%) of 132 patients had normal serum levels of procalcitonin on admission. 55 (38.2%) of 144 patients had increased serum C-reactive protein level (CRP, ≥10 mg/L). Very few patients (1.9%) had elevated cardiac troponin I (cTnI) (Table 2).

Compared to non-severe patients, severe patients presented higher percentage of shortness of breath (7.3% vs. 26.1%, P = 0.004), lower lymphocytes (median 0.8 ×10^9^/L vs. 1.2×10^9^/L, P<0.001) and albumin (ALB) levels (median 38.1 g/L vs. 41.1 g/L, P = 0.002). However, prothrombin time was not significantly different between severe patients (median 12.3 s) and non-severe patients (median 12.8 s, P = 0.089). The proportion of patients with CRP < 10mg/L, 10-50mg/L, and ≥ 50mg/L were 40.0%, 30.0%, and 30.0% in severe patients, while patients with CRP < 10mg/L, 10-50mg/L, and ≥ 50mg/L account for 65.3%, 30.6%, and 4.0% in non-severe patients, respectively. More patients showed elevated CRP in severe patients than non-severe patients (P<0.001). Furthermore, severe patients had higher fasting blood glucose (median 6.5 mmol/L) as compared with non-severe patients (median 5.7 mmol/L, P = 0.009).

**Fig 2:**
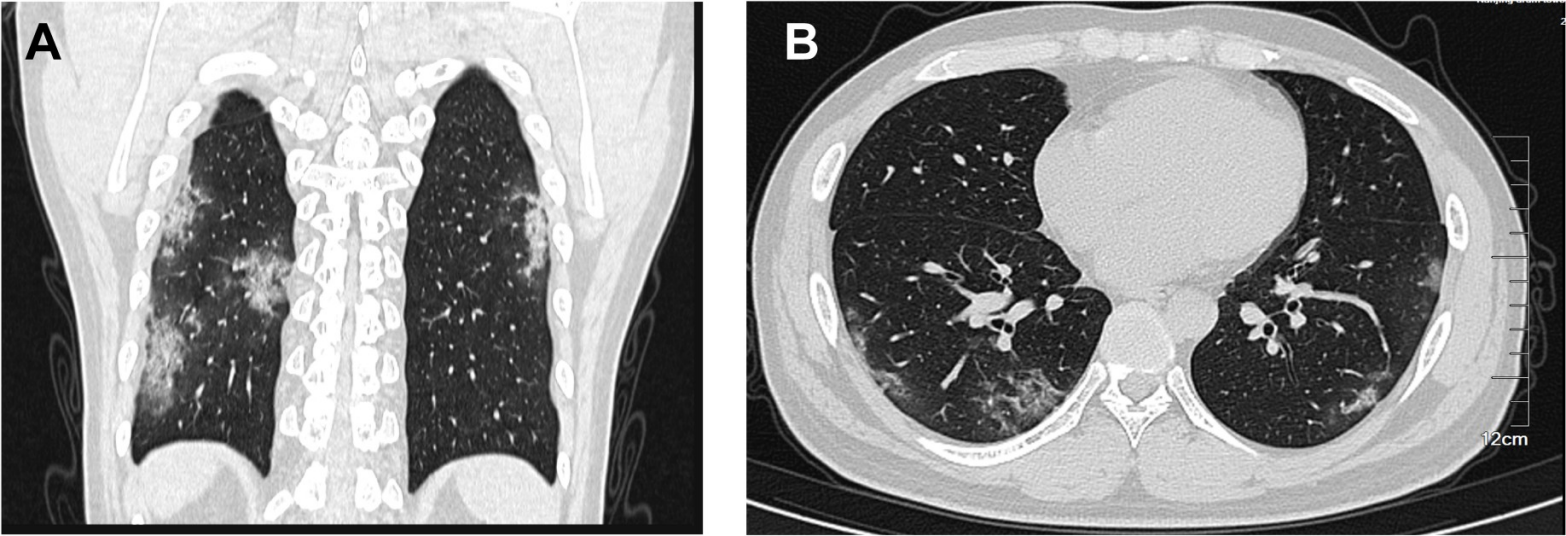
Chest computed tomographic (CT) images of a 30-year-old patient with coronavirus disease 2019 on admission. The chest CT showed bilateral ground-glass opacities in the both lungs.

On admission, abnormalities of chest CT examinations were detected in 193 (95.5%) patients. Out of 193 patients, 158 (78.2%) had bilateral involvement, and 149 (73.8%) had ground glass opacity (Table 2). Bilateral pneumonia was more commonly observed in severe patients (95.7%) than non-severe patients (76.0%, P = 0.031). Representative chest CT findings for a 30-year-old man on admission to the hospital were shown in Fig 2. Bilateral ground-glass opacities were observed in both lungs on admission (Fig 2).

### Complications, treatment and outcomes

Oxygen therapy was required in 109 (54.0%) patients and non-invasive mechanical ventilation was required in 9 (4.5%) patients. None of the patients required intubation and invasive mechanical ventilation. 196 (97.0%) patients received antiviral therapy (lopinavir/ritonavir, 89.1%; interferon α-2b, 59.9%; arbidol, 29.2%; oseltamivir, 15.8%). 149 (73.8%) patients were administered with empirical antibiotic treatment. Additionally, sixty-four (31.7%) patients were given corticosteroids and 31 (15.3%) patients were given gamma globulin. Common complications included respiratory failure (9 [4.5%] patients), ARDS (2 [1.0%] patients), and shock (1 [0.5%] patients) (Table 3). By February 10, 2020, 37 (18.3%) of 202 patients have been discharged, and 165 (81.7%) patients remained hospitalized. 11 patients were admitted to the ICU. However, no patient was deceased in our study.

**Table 3:** Treatment and short-term outcomes of patients with coronavirus disease 2019.

Treatment and outcome, n (%)	All patients (n = 202)	Non-severe patients (n = 179)	Severe patients (n = 23)	P value
**Support treatment**				
Oxygen therapy	109 (54.0)	86 (48.0)	23 (100.0)	<0.001
Nasal cannula	105 (52.0)	84 (46.9)	21 (91.3)	<0.001
Mask	20 (9.9)	6 (3.4)	14 (60.9)	<0.001
Non-invasive mechanical ventilation	9 (4.5)	0 (0)	9 (39.1)	<0.001
Invasive mechanical ventilation	0	0	0	-
**Treatment**				
Antiviral therapy	196 (97.0)	173 (96.6)	23 (100.0)	0.373
Atomized inhalation of interferon α-2b	121 (59.9)	113 (63.1)	8 (34.8)	0.009
Lopinavir/ritonavir	180 (89.1)	158 (88.3)	22 (95.7)	0.285
Arbidol	59 (29.2)	48 (26.8)	11 (47.8)	0.037
Oseltamivir	32 (15.8)	27 (15.1)	5 (21.7)	0.411
Antibiotic therapy	149 (73.8)	128 (71.5)	21 (91.3)	0.042
Use of corticosteroid	64 (31.7)	46 (25.7)	18 (78.3)	<0.001
Use of gamma globulin	31 (15.3)	21 (11.7)	10 (43.5)	<0.001
**Outcome**				
Remained in hospital	165 (81.7)	144 (80.4)	21 (91.3)	0.205
Hospital discharge	37 (18.3)	35 (19.6)	2 (8.7)	0.205
Death	0 (0)	0 (0)	0 (0)	-
Respiratory failure	9 (4.5)	0 (0)	9 (39.1)	<0.001
ARDS	2 (1.0)	0 (0)	2 (8.7)	<0.001
Shock	1 (0.5)	0 (0)	1 (4.3)	0.005
ICU admission	11 (5.4)	0 (0)	11 (47.8)	<0.001
Time from symptom onset to mechanical ventilation (days)	9.0 (8.0, 11.0)	-	9.0 (8.0, 11.0)	-

ARDS, acute respiratory distress syndrome; ICU, intensive care unit

Compared with non-severe patients, more severe patients received oxygen therapy (48.0% vs. 100.0%, P<0.001) and antibiotic therapy (71.5% vs. 91.3%, P = 0.042). Most of the severe patients received corticosteroid (78.3% vs. 25.7%, P<0.001) and more severe patients were treated with gamma globulin (43.5% vs. 11.7%, P<0.001). Regarding patients with severe illness, 11 (47.8%) patients were admitted to the ICU and 9 (39.1%) patients required non-invasive mechanical ventilation.

### Risk factors for severe coronavirus disease 2019

Univariate analysis showed that risk factors for severe illness were obesity (BMI ≥ 28 kg/m^2^), presence of type 2 diabetes, lactate dehydrogenase (LDH) > 250 U/L, CRP > 10 mg/L, and ALB < 35 g/L. However, on multivariate analysis, only the BMI ≥ 28 kg/m^2^ (odds ratio [OR], 9.219; 95% confidence interval [CI], 2.731 to 31.126; P<0.001) and a known history of diabetes (OR, 4.326; 95% CI, 1.059 to 17.668; P = 0.041) were the independent risk factors for developing severe illness (Table 4).

**Table 4:** Univariate and multivariate analysis of risk factors for severe coronavirus disease 2019.

Variables	Univariate		Multivariate	
	OR (95%CI)	P-value	OR (95%CI)	P value
Age (yr)				
≤60	Reference			
>60	2.090 (0.702, 6.218)	0.185		
Gender				
Female	Reference			
Male	2.290 (0.863, 6.078)	0.096		
BMI (kg/m^2^)				
Non-obesity (<28)	Reference			
Obesity (≥28)	6.900 (2.381, 19.997)	<0.001	9.219 (2.731, 31.126)	<0.001
Hypertension				
No	Reference			
Yes	0.536 (0.119, 2.420)	0.418		
Type 2 diabetes				
No	Reference			
Yes	8.145 (2.842, 23.342)	<0.001	4.326 (1.059, 17.668)	0.041
Smoking				
No	Reference			
Yes	1.109 (0.235, 5.223)	0.896		
WBC (×10^9^/L)				
No decreased	Reference			
Decreased	0.886 (0.331, 2.374)	0.809		
Neutrophils (×10^9^/L)				
No decreased	Reference			
Decreased	0.576 (0.162, 2.042)	0.393		
Lymphocyte (×10^9^/L)				
No decreased	Reference			
Decreased	2.067 (0.859, 4.971)	0.101		
Hb (g/L)				
>120	Reference			
≤120	0.717 (0.157, 3.277)	0.667		
PLT (×10^9^/L)				
>100	Reference			
≤100	2.291 (0.589, 8.910)	0.232		
ALT (U/L)				
≤40	Reference			
>40	2.100 (0.750, 5.879)	0.158		
LDH (U/L)				
≤250	reference			
>250	4.151 (1.444, 11.932)	0.008		
Tbil (μmol/L)				
≤20	Reference			
>20	1.917 (0.503, 7.310)	0.341		
ALB (g/L)				
>35	Reference			
≤35	4.600 (1.542, 13.722)	0.006		
CR (μmol/L)				
≤90	Reference			
>90	1.759 (0.535, 5.779)	0.352		
CRP (mg/L)				
<10	Reference			
≥10	2.826 (1,073, 7.439)	0.035		
PT (s)				
≤14.0	Reference			
>14.0	0.507 (0.062, 3.961)	0.507		

BMI, body mass index; WBC, white blood cells; Hb, hemoglobin; PLT, platelet; ALT, alanine transaminase; LDH, lactate dehydrogenase; Tbil, total bilirubin; ALB, albumin; CR, creatinine; CRP, C-reactive protein; PT, prothrombin time; OR, odds ratio; CI, confidence interval.

## Discussion

Our study provides a comprehensive description of the clinical characteristics of laboratory-confirmed cases of COVID-19, and the risk factors for severe COVID-19 in 202 cases from 8 designated hospitals in 8 cities of Jiangsu province, China.

Consistent with the study by Wang et al., about half of the patients in our study were male [7]. These data differ from the recent reports by Huang et al. and Chen et al. which showed SARS-CoV-2 is more likely to infect male [4,6]. One possible explanation is that more COVID-19 patients in the previous report had an exposure history of the Huanan Seafood Market in Wuhan, and most of patients tended to be male workers [7]. However, only 1.5% of patients in our study had known contact with Huanan Seafood Market. The median age of the patients was 44 years and only 12.9% of the patients aged over 60 years. 27.2% of the patients had chronic comorbidities. Several studies suggested that SARS-CoV-2 is more likely to infect elder adult males with chronic comorbidities [4,7,17]. However, our study indicated that patients at a wide age range can be infected by SARS-CoV-2. Recently, Wei et al. even reported nine infants infected by SARS-CoV-2 [18]. Although 15.8% of the patients did not report any known contact with Wuhan-related people, the majority of patients in our study were Wuhan-related which indicated the epidemiology are important for the diagnosis of COVID-19 for patients outside Wuhan.

Consistent with two recent reports, fever and cough were the common symptoms whereas other symptoms such as diarrhea were much rare [4,6]. However, our study found that 22.8% of the patients were afebrile on admission. The percentage of patients with fever was lower than the previous study which was ranged from 83% to 98.6% [7]. Thus, the afebrile patients with an epidemiological link of the disease should be also suspected for COVID-19. Compared with symptoms of non-severe patients, shortness of breath was more common in severe patients. The most common laboratory abnormalities observed in this study were decreased white blood cells and lymphocyte counts as well as increased CRP and LDH levels. Compared to non-severe patients, severe patients had laboratory abnormalities such as lower lymphocytes and ALB levels as well as higher LDH and CPR levels. The onset of symptoms and laboratory abnormalities on admission may help the physicians identify the COVID-19 patients who likely develop severe illness and provide better supportive care.

Currently, except for meticulous supportive care, no specific treatment has been recommended for COVID-19. Although antibacterial agents are ineffective for COVID-19, over 70% of the patients in this study still receive antibacterial agents. About 90% of the patients received antiviral therapy, such as atomized inhalation of interferon α-2b, lopinavir/ritonavir, arbidol, and oseltamivir. The benefit of the antiviral agents for COVID-19 is not yet clear and deserves further investigation. 31.7% of the patients used corticosteroids during the treatment, especially for severe patients (78.3%). Although some experts recommend the prudently use of short courses of corticosteroids at low-to-moderate doses for critically ill patients with COVID-19, the use of corticosteroids remains controversial in COVID-19 [19]. Moreover, the optimal time, dose and duration of corticosteroids for patients with COVID-19 are not yet clear and need to be evaluated by randomized controlled trials to provide a more solid evidence for treatment recommendations in the future. Gamma globulin was also used for the treatment of COVID-19 and severe patients were more likely to receive gamma globulin treatment. However, the efficacy of gamma globulin for COVID-19 remains controversial.

23 (11.4%) patients were identified as severe illness cases in our study which is lower than the previous study (15.7%) [20]. The ICU admission (5.4%) was also lower as compared with the previous studies which ranged from 26.1% to 32% [6,7]. As of February 10, 2020, no patients died in our study. The mortality rate in our study is significantly lower than patients in Wuhan as previously reported which ranged from 4.3% to 15% [6,7]. Wu et al. also reported only 1 of 62 patients was transferred to an ICU and no patient died in Zhejiang province [9]. The possible interpretation of the mild disease and better outcomes in our study may be that the patients were younger than previous reports. The elderly people may have an increased risk of coexisting disorders and are more susceptible to developing severe forms of disease than younger people [21,22]. Indeed, less patients had comorbidities (27.2%) in our study as compared with the previous reports (32.0%-46.4%) [4,6,7]. The median time from symptom onset to admission was 5.0 days which is also shorter than the previous studies (7.0 days) [6,7]. Thus, early diagnosis and prompt care of the COVID-19 patients might also together contribute to the significant reduction in mortality rate in our study [20].

Early identification of patients with severe illness is important for the management of patients with COVID-19. We conducted univariate and multivariate analysis for risk factors of COVID-19 patients with severe illness on admission. For the first time, we identified the obesity (BMI over 28 kg/m^2^) and a history of type 2 diabetes as two independent risk factors of severe illness, suggesting that the metabolic conditions was associated the severity of COVID-19. In the previous study, metabolic syndrome-related conditions such as diabetes and obesity were also demonstrated to be etiologically linked to middle east respiratory syndrome coronavirus (MERS-CoV) pathogenesis [23]. These disorders were reported to down regulate some key mediators of host immune response to pathogenesis [23,24]. Previous study found that a known history of type 2 diabetes was also an independent predictor of death and morbidity in patients with severe acute respiratory syndrome (SARS) [25]. Whether the obesity and known history of type 2 diabetes could predict the fatal outcome of patients with COVID-19 deserves further investigation.

This study has several limitations. First, only 202 patients with confirmed COVID-19 were included from eight cities of Jiangsu province. It would be better to include as many as possible patients in Jiangsu province to get more comprehensive understanding of COVID-19 outside Wuhan. Moreover, we only included patients with laboratory confirmed cases. The suspected but undiagnosed cases were not included in the present study. As of February 10, 2020, 515 confirmed cased have been reported in Jiangsu province [5]. In fact, about a half of the confirmed cases were included in our present study. Thus, our study is very representative. Second, clinical outcomes of the remaining 165 hospitalized patients were not available at the time of analysis. Thus, we could not assess the risk factors for poor outcomes of patients. However, this multicenter study provides an early assessment of the epidemiological and clinical characteristics of COVID-19 outside Wuhan, China.

In conclusion, COVID-19 patients in Jiangsu province were less severe and have improved prognosis than the initial COVID-19 patients as previously reported in Wuhan indicating that mild cases might already have occurred. A BMI > 28 kg/m^2^ and a history of type 2 diabetes are independent risk factors for severe illness of COVID-19. Sustained intensive control efforts remain urgently needed to minimize the risk of COVID-19.

## References

[1] Zhu N, Zhang D, Wang W, Li X, Yang B, Song J, et al A Novel Coronavirus from Patients with Pneumonia in China, 2019. *N Engl J Med*. 2020;382(8):727–733. 10.1056/NEJMoa2001017 31978945 PMC7092803

[2] Gorbalenya AE, Baker SC, Baric RS, Groot RJ, Drosten C, Gulyaeva AA, et al Severe acute respiratory syndrome-related coronavirus: The species and its viruses–a statement of the Coronavirus Study Group. *BroRxiv*. 2020 [published on Feb 11, 2020]. 10.1101/2020.02.07.937862

[3] Wang C, Horby PW, Hayden FG, Gao GF. A novel coronavirus outbreak of global health concern. *Lancet*. 2020;395(10223):470–473. 10.1016/S0140-6736(20)30185-9 31986257 PMC7135038

[4] Chen N, Zhou M, Dong X, Qu J, Gong F, Han Y, et al Epidemiological and clinical characteristics of 99 cases of 2019 novel coronavirus pneumonia in Wuhan, China: a descriptive study. *Lancet*. 2020;395(10223):507–513. 10.1016/S0140-6736(20)30211-7 32007143 PMC7135076

[5] World Health Organization. Novel Coronavirus (COVID-19) Situation. https://experience.arcgis.com/experience/685d0ace521648f8a5beeeee1b9125cd. Accessed March 13, 2020.

[6] Huang C, Wang Y, Li X, Ren L, Zhao J, Hu Y, et al Clinical features of patients infected with 2019 novel coronavirus in Wuhan, China. *Lancet* 2020;395(10223):497–506. 10.1016/S0140-6736(20)30183-5 31986264 PMC7159299

[7] Wang D, Hu B, Hu C, Zhu F, Liu X, Zhang J, et al Clinical Characteristics of 138 Hospitalized Patients With 2019 Novel Coronavirus-Infected Pneumonia in Wuhan, China. [Published Feb 7, 2020]. *JAMA*. 2020 10.1001/jama.2020.1585 32031570 PMC7042881

[8] Chang D, Lin M, Wei L, Xie L, Zhu G, Dela Cruz CS, et al Epidemiologic and Clinical Characteristics of Novel Coronavirus Infections Involving 13 Patients Outside Wuhan, China. [Published Feb 7, 2020]. *JAMA*. 2020 10.1001/jama.2020.1623 32031568 PMC7042871

[9] Xu X, Wu X, Jiang X, Xu KJ, Ying LJ, Ma CL, et al Clinical findings in a group of patients infected with the 2019 novel coronavirus (SARS-Cov-2) outside of Wuhan, China: retrospective case series. [Published Feb 19, 2020]. *BMJ*. 2020 10.1136/bmj.m606 32075786 PMC7224340

[10] World Health Organization. Clinical management of severe acute respiratory infection when novel coronavirus (nCoV) infection is suspected: interim guidance. Published January 28, 2020. Accessed January 31, 2020. https://www.who.int/publications-detail/clinical-managementof-severe-acute-respiratory-infection-when-novelcoronavirus-(ncov)-infection-is-suspected.

[11] ARDS Definition Task Force, Ranieri VM, Rubenfeld GD, Thompson BT, Ferguson ND, Caldwell E, et al Acute respiratory distress syndrome: the Berlin Definition. *JAMA*. 2012;307(23):2526–2533. 10.1001/jama.2012.5669 22797452

[12] Khwaja A. KDIGO clinical practice guidelines for acute kidney injury. *Nephron Clin Pract*. 2012;120(4):c179–184. 10.1159/000339789 22890468

[13] Thawley V. Acute Liver Injury and Failure. *Vet Clin North Am Small Anim Pract*. 2017;47(3):617–630. 10.1016/j.cvsm.2016.11.010 28065578

[14] Gao C, Wang Y, Gu X, Shen X, Zhou D, Zhou S, et al Association Between Cardiac Injury and Mortality in Hospitalized Patients Infected With Avian Influenza A (H7N9) Virus. [published January 20, 2020].*Crit Care Med*. 2020 10.1097/CCM.0000000000004207 32205590 PMC7098447

[15] National Health Commission. Guidelines for the Diagnosis and Treatment of Novel Coronavirus (2019-nCoV) Infection by the National Health Commission (Trial Version 5). (http://www.nhc.gov.cn/yzygj/s7653p/202002/3b09b894ac9b4204a79db5b8912d4440.shtml) (accessed Feb 16, 2020). 10.3760/cma.j.issn.0376-2491.2020.0001 32033513

[16] Laboratory diagnostics for novel coronavirus. WHO 2020 (https://www.who.int/health-topics/coronavirus/laboratory-diagnostics-for-novel-coronavirus) (accessed Feb 6, 2020).

[17] Li Q, Guan X, Wu P, Wang X, Zhou L, Tong Y, et al Early Transmission Dynamics in Wuhan, China, of Novel Coronavirus-Infected Pneumonia. [published Jan 29, 2020]. *N Engl J Med*. 2020 10.1056/NEJMoa2001316 31995857 PMC7121484

[18] Wei M, Yuan J, Liu Y, Fu T, Yu X, Zhang ZJ. Novel Coronavirus Infection in Hospitalized Infants Under 1 Year of Age in China. [published Feb 14, 2020]. *JAMA*. 2020 10.1001/jama.2020.2131 32058570 PMC7042807

[19] Shang L, Zhao J, Yi H, Du R, Cao B. On the use of corticosteroids for 2019-nCoV pneumonia. [Published Feb 19, 2020]. *Lancet*. 2020 10.1016/S0140-6736(20)30361-5 32122468 PMC7159292

[20] Guan WJ, Ni ZY, Hu Y, Liang WH, Ou CQ, He JX, et al Clinical characteristics of 2019 novel coronavirus infection in China. N Engl J Med. 2020 2 28 10.1056/NEJMoa2002032 32109013 PMC7092819

[21] Hanshaoworakul W, Simmerman JM, Narueponjirakul U, Sanasuttipun W, Shinde V, Kaewchana S, et al Severe human influenza infections in Thailand: oseltamivir treatment and risk factors for fatal outcome. *PloS One*. 2009;4(6):e6051 10.1371/journal.pone.0006051 19557130 PMC2699035

[22] Vaccaro JA, Gaillard T, Huffman FG, Vieira ER. Motivational Strategies to Prevent Frailty in Older Adults with Diabetes: A Focused Review. *J Aging Res*. 2019;2019:3582679 10.1155/2019/3582679 31885920 PMC6893277

[23] Badawi A, Ryoo SG. Prevalence of comorbidities in the Middle East respiratory syndrome coronavirus (MERS-CoV): a systematic review and meta-analysis. *Int J Infect Dis*. 2016;49: 129–133. 10.1016/j.ijid.2016.06.015 27352628 PMC7110556

[24] Odegaard JI, Chawla A. Connecting type 1 and type 2 diabetes through innate immunity. *Cold Spring Harb Perspect Med*. 2012;2(3):a007724 10.1101/cshperspect.a007724 22393536 PMC3282495

[25] Yang JK, Feng Y, Yuan MY, Yuan SY, Fu HJ, Wu BY, et al Plasma glucose levels and diabetes are independent predictors for mortality and morbidity in patients with SARS. *Diabet Med*. 2006;23(6):623–628. 10.1111/j.1464-5491.2006.01861.x 16759303

